# MiR-208b Regulates the Conversion of Skeletal Muscle Fiber Types by Inhibiting Mettl8 Expression

**DOI:** 10.3389/fgene.2022.820464

**Published:** 2022-02-23

**Authors:** Xiang Li, Hanfang Bi, Shanshan Xie, Wentao Cui

**Affiliations:** Institute of Animal Sciences, Chinese Academy of Agricultural Sciences, Beijing, China

**Keywords:** skeletal muscle, muscle fiber type conversion, miR-208b, Mettl8, miRNA, myostatin

## Abstract

Skeletal muscle, the main source of animal meat products, contains muscle fiber as a key unit. It is well known that transformation takes place between different types of muscle fibers, however, the conversion mechanism is not clear. In a previous study, our lab has demonstrated that there is a decrease in type I muscle fibers and an increase in type IIB muscle fibers in skeletal muscle of myostatin gene-edited Meishan pigs. Very interestingly, we observed the down regulation of miR-208b expression and an increase in expression the predicted target gene Mettl8 (Methyltransferase like 8) in skeletal muscle of MSTN gene-edited Meishan pigs. These results reveal that there is a potential connection between the conversion of skeletal muscle fiber types and miR-208b and Mettl8 expression. In this study, we first explored the expression patterns of miR-208b and Mettl8 in skeletal muscle in Meishan pigs; and then C2C12 cells were used to simulate the development and maturation of muscle fibers. Our results indicated that Myh4 expression level decreased and Myh7 expression level increased following overexpression of miR-208b in C2C12 cells. We therefore speculate that miR-208b can promote the conversion of fast-twitch fibers to slow-twitch fibers. The targeting relationship between Mettl8 and miR-208b was confirmed by results obtained using dual luciferase assay, RT-qPCR, and WB analysis. Following the transfection of Mettl8 siRNA into C2C12 cells, we observed that Mettl8 expression decreased significantly while Myh7 expression increased and Myh4 expression decreased, indicating that Mettl8 promotes the conversion of slow muscle fibers to fast muscle fibers. Additionally, changes in skeletal muscle fiber types are observed in those mice where miR-208b and Mettl8 genes are knocked out. The miR-208b knockout inhibits the formation of slow muscle fibers, and the Mettl8 knockout inhibits the formation of fast muscle fibers. In conclusion, our research results show that miR-208b regulates the conversion of different muscle fiber types by inhibiting Mettl8 expression.

## Introduction

Skeletal muscle accounts for 30–40% of body weight in mammals. It is the main source of meat products for meat-producing animals and also an important metabolic organ (Doran et al., 2009; Listrat et al., 2016; Abel, 2018). Skeletal muscle dysfunction is related to a variety of muscle diseases, such as sarcopenia, muscle hypertrophy, amyotrophic lateral sclerosis, and muscle atrophy in diabetic patients (Fontelonga et al., 2019; Garneau and Aguer, 2019; Deldicque, 2020). There are four types of skeletal muscle fibers: slow contraction oxidative metabolism type (I), fast contraction oxidative metabolism type (IIA), fast contraction glycolytic metabolism type (IIB), and fast contraction oxidative glycolysis facultative metabolism type (IIX). Different composition of muscle fiber types in skeletal muscle is a key factor that determines meat quality (Choi et al., 2007). For example, a high proportion of slow-oxidized muscle fibers improves meat tenderness, juiciness, and color (Lee et al., 2010).

Muscle-related genes such as myogenic regulatory factors (MRFs) and myostatin (MSTN) play important role in regulation of composition of different muscle fiber types. Hennebry et al. (Hennebry et al., 2009) demonstrated that loss of MSTN through gene knockout method resulted in an increase in type IIB muscle fibers and a decrease in type IIA and type I muscle fibers in mouse tibial anterior muscle. In recent years, non-coding RNAs have been shown to be involved in the regulation of muscle fiber transformation. Long-chain non-coding RNAs lnc-Six1 (Tieland et al., 2018) and lnc-mg (Du et al., 2019) can be used as miRNA molecular sponges to indirectly affect the expression of related genes, thereby regulating the transformation between fast and slow muscle fibers. In addition, miRNAs also play a key role in the regulatory network of muscle fiber typing by regulating the expression of target genes.

Micro RNAs (miRNAs) are a class of highly conserved single-stranded, non-coding RNAs with a length of 21–24 base pairs (Bhat et al., 2016; Lu and Rothenberg, 2018). MiRNAs play their regulatory roles by directly degrading target genes or by inhibiting target gene translation. MiR-208b, a member of miR-208 family, is encoded by the introns of the *β*-cardiac myosin heavy chain protein gene Myh7 and is specifically expressed in myocardium and skeletal muscle (Van Rooij et al., 2008). Recent studies on miR-208b focus mainly on heart disease, but miR-208b’s specific mechanism of action to regulate skeletal muscle growth and development is still unclear. Additionally, Methyltransferase like 8 (Mettl8) is a member of the methyltransferase-like protein family (Badri et al., 2008; Tobi et al., 2018), which is mainly involved in cell differentiation, cell component formation, protein metabolism, and phylogenetic processes. Although Mettl8 is widely expressed, there is no report on the specific role of Mettl8 in skeletal muscle growth and development.

Our lab recently produced genetically engineered Meishan pigs containing a ZFN-edited myostatin loss-of-function MKO mutation (MSTN^−/−^) that led to the hypertrophy of skeletal muscles (Qian et al., 2015). Analysis of deep miRNA sequencing data from skeletal muscle predicated that the expression of miR-208b was down-regulated and expression of miR-208b’s target gene Mettl8 was up-regulated in MSTN^−/−^ pigs (Xie et al., 2019; Li et al., 2020), implying that miR-208b and Mettl8 may play roles in skeletal muscle growth and development and regulating function of MSTN. We also measured the changes in the muscle fiber composition of the longissimus dorsi muscle in MSTN^−/−^ Meishan pigs. Results indicated that type I fibers were reduced while type IIB fibers increased (Qian et al., 2015), suggesting that the changes in skeletal muscle fiber types in Meishan pigs induced by MSTN knockout may be related to miR-208b and its target gene Mettl8. Therefore, in this study, we first analyzed the expression patterns of miR-208b and Mettl8 in Meishan pigs, and then analyzed the effect of miR-208b on MyHC gene expression during the differentiation process of C2C12 cells by either overexpressing or inhibiting miR-208b. Bioinformatics analysis methods, dual luciferase reporter assay system and RNA interference experiment were used to predict and verify the target gene Mettl8 of miR-208b, and initially explore the effect of Mettl8 on the skeletal muscle fiber transformation process. Finally, we generated miR-208b and Mettl8 gene knockout mice to further analyze its function in the process of skeletal muscle fiber transformation in mice. Our research on the law of muscle fiber transformation may provide a new direction for the selection of excellent varieties and the treatment of skeletal muscle diseases.

## Materials and Methods

### Animals

Both MKO (MSTN^−/−^) and MWT (MSTN wild type, MSTN^+/+^) Meishan pigs were produced using zinc finger nucleases (ZFN) and somatic cell nucleus transfer (SCNT) techniques (Qian et al., 2015). All pigs were maintained in Qingdao animal facility, fed with the same standard diet, and raised under the same conditions. 65 days of embryo development, 4-month old, 8-month-old, 16-month-old male pigs were used in this study. The gene-edited C57BL/6 mice were prepared by the Institute of Zoology, Chinese Academy of Sciences using CRISPR/Cas9 technology and microinjection technology. All experimental mice were 8 weeks old and weighed about 22 g. All samples were quickly collected after the animals were euthanized. The tissues used to extract total RNA or protein were quickly frozen in liquid nitrogen, and the tissues used for paraffin sections were immersed in 4% tissue fixative.

### Cell Culture and Transfection

The HEK293T cells used for dual luciferase reporter assay were preserved by our laboratory. C2C12 myoblast cells were purchased from the Cell Resource Center in IBMS in CAMS/PUMC. The cells were cultured in growth medium consisting of Dulbecco’s modified Eagle’s medium (DMEM) supplemented with 10% FBS and 1% penicillin/streptomycin. Myogenic differentiation was induced by replacing differentiation medium (DMEM supplemented with 2% horse serum and 1% penicillin/streptomycin) when cell confluence reached 60–70%.

Transfection was performed with the Lipofectamine 2000 reagent (Invitrogen) combined with 50 nM miRNA mimics or corresponding control, 100 nM miRNA inhibitor or corresponding control, 50 nM Mettl8 siRNA or corresponding control when cell confluence reached 70–80%.

### Plasmids Construction

The region of Mettl8 3′ UTR flanking the miR-208b binding site was amplified from mouse genomic DNA using PCR. The target sequence GGGAGCT (800–806 bp) was mutated to CCCTCGA using overlap PCR. Primers were showed in Supplementary Table S1. The PCR product was cloned into the vector downstream of the Renilla Luciferase open reading frame using the NotI and XhoI restriction sites. We obtained two pmiR-RB-REPORT vectors (RiboBio) with wide-type and mutant 3′ UTR of Mettl8.

### Dual Luciferase Reporter Assay

HEK293T cells were co-transfected with 100 ng of the wide-type or mutant 3′UTR luciferase reporter and 50 nM of the miR-208b mimics or control duplexes using the Lipofectamine 2000 reagent (Invitrogen) in 48-well plates. After transfection for 48 h, cells were harvested by adding 300 μL of a passive lysis buffer. Renilla and firefly luciferase activities were measured with the Dual Luciferase Assay System (Promega, Madison, WI) in a TD-20/20 luminometer (Turner Biosystems, Sunnyvale, CA), and the Renilla luciferase signal was normalized to the firefly luciferase signal. The normalized Renilla luciferase activity was compared with the wild type, miR-208b, and the mutant using the Student’s *t* test (*p* < 0.05).

### RNA Isolation and Quantitative Real-Time PCR

Total RNA was extracted from muscle samples by using TRIzol (Invitrogen) per manufacturer’s instructions. RNA quality was assessed by using the RNA Nano 6000 Assay Kit of the Bioanalyzer 2100 system (Agilent Technologies, CA, United States), agarose gel electrophoresis and NanoDrop (Thermo Fisher). Each sample (1 μg) was reverse transcribed into cDNA by using the RevertAid^TM^ First Strand cDNA Synthesis Kit (Fermentas). Real-time PCR was performed in Quant Studio 3 system (Thermo Fisher ABI) using 10 pM of each specific primer (Supplementary Table S1) and SYBR Premix ExTagTM (Takara) according to the manufacturer’s protocols. The 2-^ΔΔCt^ method was employed to calculate the relative expression levels of mRNAs (Livak and Schmittgen, 2001).

### Immunoblotting

For protein extraction, 2 mL of lysis buffer [8 mol/L urea, 2% SDS, × 1 Protease Inhibitor Cocktail (Roche Ltd. Basel, Switzerland)] was added to each sample, followed by sonication on ice and centrifugation at 13,000 rpm for 10 min at 4°C. The supernatant was then transferred to a fresh tube. Total protein concentration was determined using a BCA Quantitative Test Kit (Beyotime). Each protein sample was loaded in equal amount and then separated by 10% or 12.5% SDS PAGE. Following transfer of protein from gel to nitrocellulose (NC) membrane and blocked with 5% skimmed milk for 2 h, immunoblotting was performed using standard method for the following proteins with corresponding detection antibodies: Mettl8 (Rabbit polyclonal antibody, Biorbyt), Myh4 (Monoclonal Antibody, Thermo Scientific), Myh7 (Rabbit polyclonal antibody, Santa Cruz), myosin light chain 9 (MYL9) (Rabbit polyclonal antibody, Abcam), beta-tublin (Rabbit polyclonal antibody, Cell Signaling Technology), beta-actin (Rabbit polyclonal antibody, Abcam). Beta-tublin and beta-actin were used as an internal reference in Western blot. Super Signal West Pico chemiluminescent substrate (Thermo Fisher Scientific) was used to develop color band. Image J software was used to analyze protein band density. Graphd Prism 8.0 software was used to make histograms using protein band density data.

### Haematoxylin and Eosin (HE) Staining

The gastrocnemius (GAS) and soleus muscle (SOL) were dissected at the time when mice were euthanized, fixed in 4% paraformaldehyde, and embedded in paraffin. Muscle sections were stained with hematoxylin and eosin, and pictures were taken from four random fields at ×40 magnification. HE staining method are the same as previously described (Cai et al., 2017).

### Statistical Analysis

The data of all experimental groups and control groups were statistically analyzed using the Analyze procedure in Graphd Prism 8.0 software. All data were expressed as average ±standard deviation. Unpaired *t*-test, multiple *t*-test (Multiple comparisons between two samples)or one-way variance test (comparison between multiple groups) was used to identify if there was differential expression. *p* < 0.05 is considered statistically significant.

## Results

### Expression Patterns of miR-208b and Mettl8 in Meishan Pigs

**FIGURE 1:**
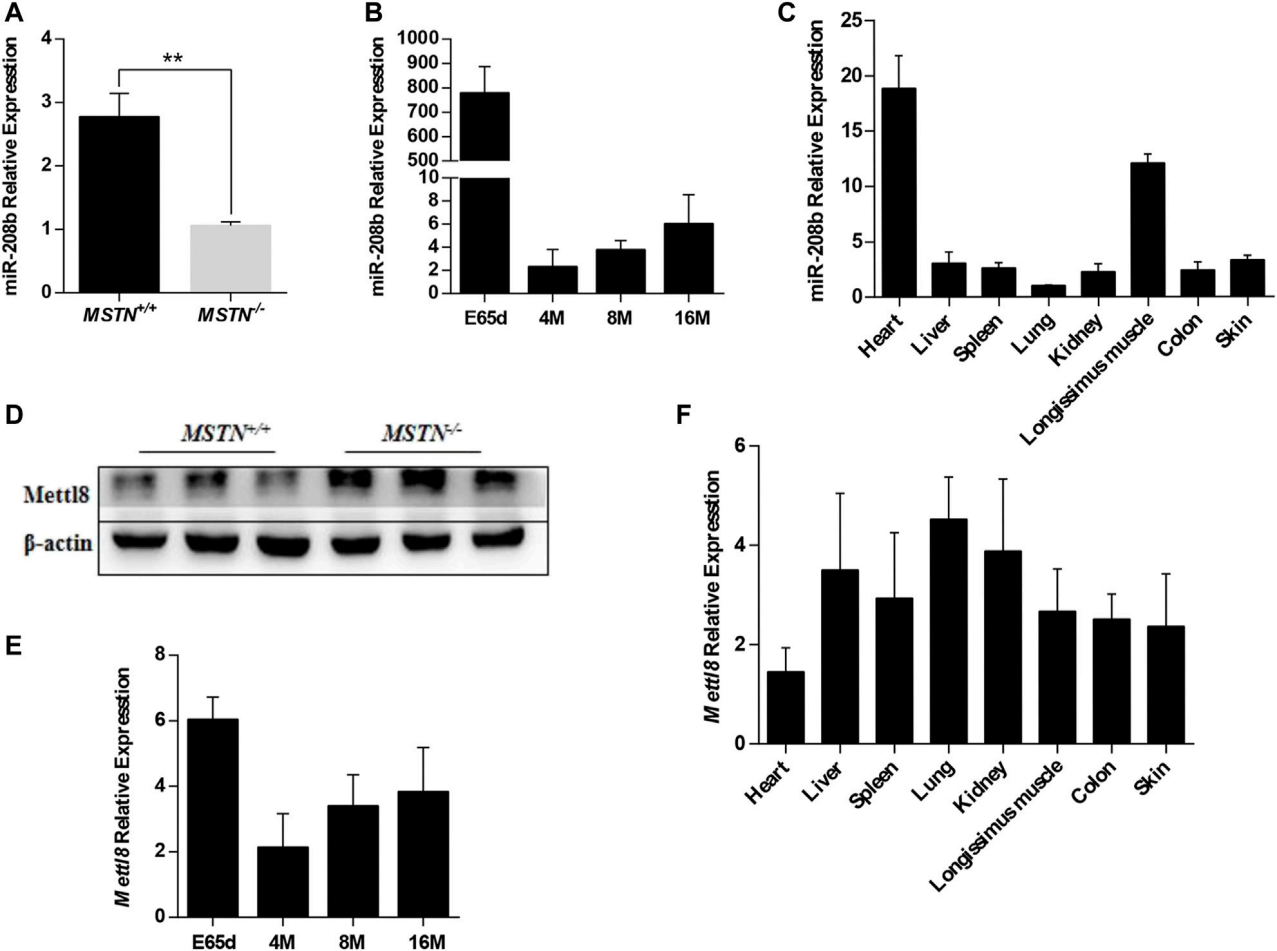
Expression pattern of miR-208b and Mettl8 in Meishan pigs. **(A)** Expression level of miR-208b in the longissimus dorsi from wild type (MSTN^+/+^) and MSTN^−/-^ Meishan pigs on E65d. **(B)** Expression level of miR-208b in the longissimus dorsi from wild type (MSTN^+/+^) Meishan pigs at different ages. **(C)** Expression level of miR-208b in different tissues from wild type (MSTN^+/+^) Meishan pigs on E65d. **(D)** Expression level of Mettl8 protein in the longissimus dorsi muscle from wild type (MSTN^+/+^) and MSTN^−/-^ Meishan pigs on E65d. **(E)** Expression level of Mettl8 protein in the longissimus dorsi muscle from wild type (MSTN^+/+^) at different ages. **(F)** Expression level of Mettl8 protein in different tissues from wild type (MSTN^+/+^) on E65. E65d means pigs on day 65 of embryonic development, 4, 8, 16 M indicate 4, 8, and16 months after birth, respectively. Statistical comparisons are performed using unpaired *t*-tests in **(A,B)** (The data of 4, 8 and 16 M are compared with the data of E65d, respectively), **(E)** (The data of 4, 8 and 16 M are compared with the data of E65d, respectively)and using ordinary one-way ANOVA in **(C,F)**. **p < 0.01.

We have found that Mettl8 and miR-208b were related to each other by multi-omics analysis of MSTN gene-edited Meishan pig skeletal muscle (Xie et al., 2019; Li et al., 2020). Sequencing results showed that compared with MSTN^+/+^ group, the expression level of miR-208b was significantly down-regulated while the expression level of Mettl8, was significantly up-regulated in MSTN^−/−^ group (Xie et al., 2019; Li et al., 2020). In this study, the results of RT-qPCR and Western blot were consistent with previous sequencing results (Figures 1A,D). We tested miR-208b expression profiles during growth different stages and in different tissues in Meishan pigs (Figures 1B,C). The results showed that miR-208b had the highest expression in skeletal muscle on 65 days of embryo development but decreased post birth. It is speculated that miR-208b mainly plays a role in the embryonic stage of Meishan pigs. The expression level of miR-208b is the highest in the myocardium and skeletal muscle of Meishan pigs, indicating that miR-208b is a muscle-specific miRNA and plays its role mainly in the striated muscle of Meishan pigs. We also further tested Mettl8 expression profiles during growth different stages and in different tissues in Meishan pigs (Figures 1E,F). The results showed that Mettl8 expression level in the longissimus dorsi of Meishan pigs is higher on 65 days of embryo development in other growth periods. Although Mettl8 is widely expressed in a variety of tissues, its expression in myocardium and skeletal muscle is relatively low, indicating that it may play an opposite role when compared to miR-208b.

### MiR-208b Promotes the Conversion of Fast Muscle Fibers to Slow Muscle Fibers During C2C12 Myogenic Differentiation

**FIGURE 2:**
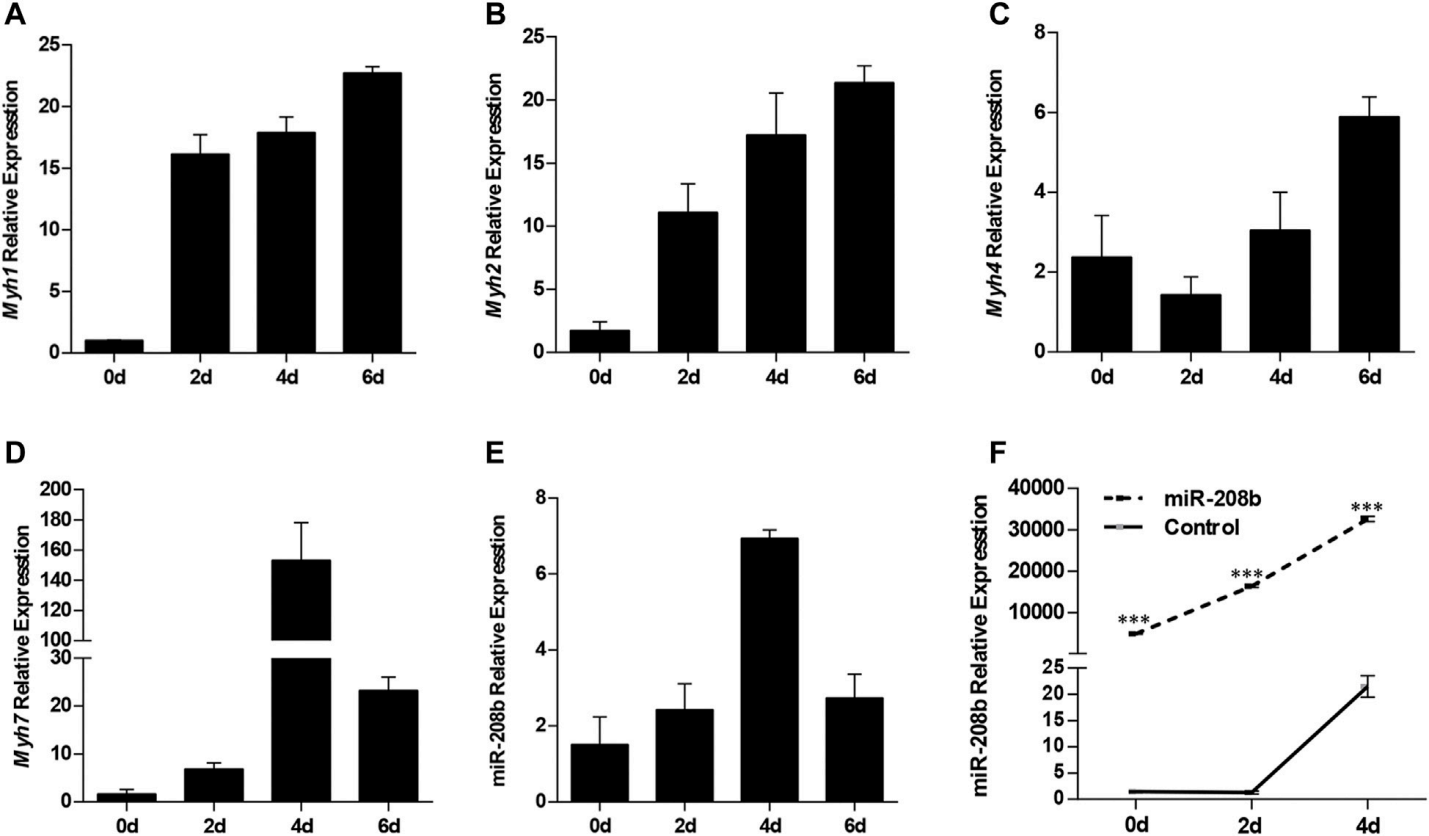
The expression patterns of MyHC and miR-208b during of C2C12 cell myogenic differentiation. **(A–E)**: Expression levels of Myh1 **(A)**, Myh2 **(B)**, Myh4 **(C)**, Myh7 **(D)**, and miR-208b **(E)** during C2C12 myogenic differentiation on days 0, 2, 4, and 6. **(F)** Relative expression level of miR-208b during C2C12 myogenic differentiation on days 0, 2, and 4 post transfections with miR-208b mimics and control vectors, respectively. Statistical comparisons are performed using ordinary one-way ANOVA in **(A–E)** and using multiple *t*-tests in **(F)**.

**FIGURE 3:**
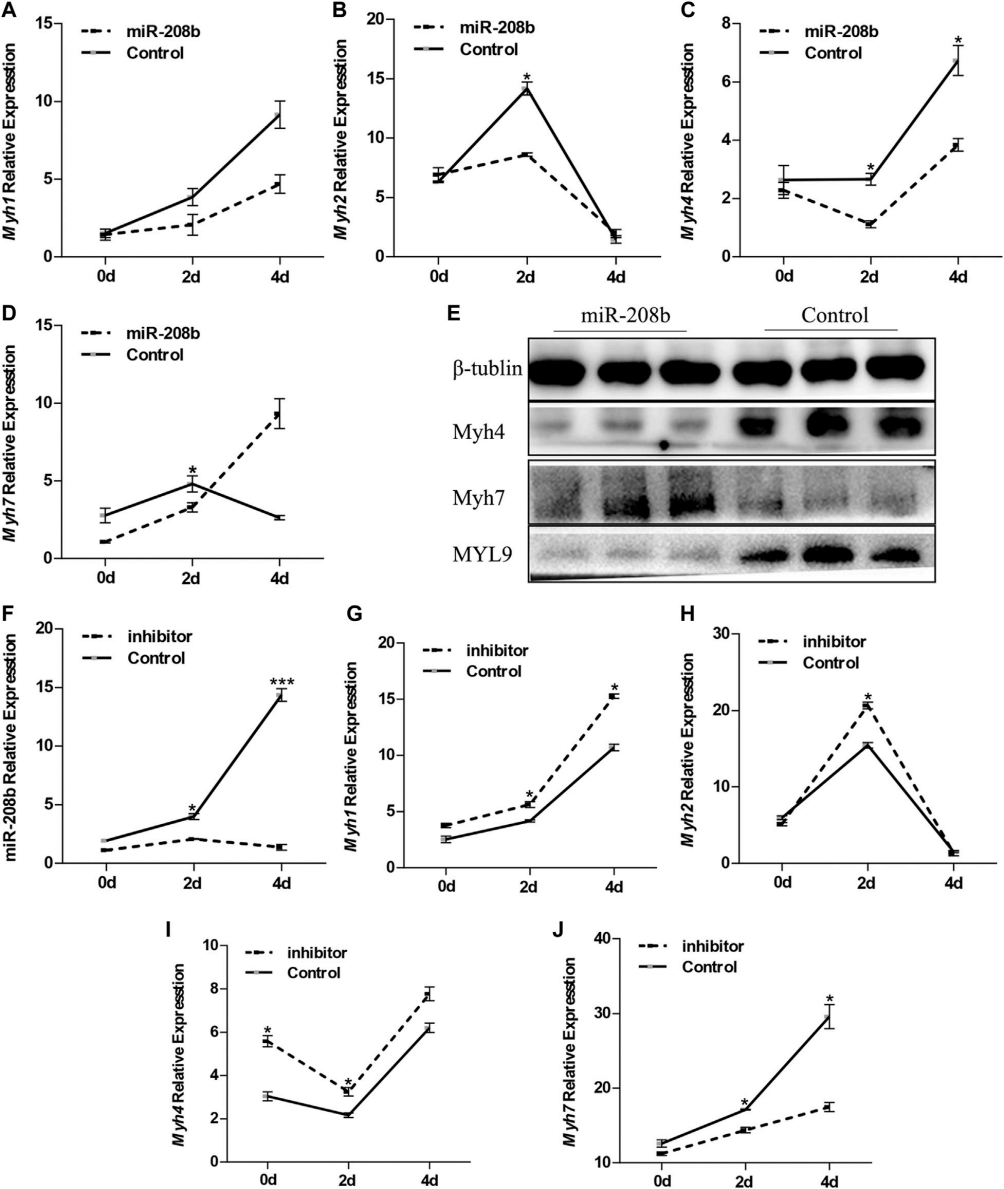
Effect of miR-208b on MyHC gene expression during myogenic differentiation. **(A–D)**: relative mRNA expression levels of Myh1 **(A)**, Myh2 **(B)**, Myh4 **(C)**, and Myh7 **(D)** in C2C12 cells overexpressing miR-208b on days 0, 2, 4 of myogenic differentiation. **(E)** protein expression levels of Myh4, Myh7, MYL9 post 4 days of miR-208b over-expression during C2C12 myogenic differentiation. miR-208b represents the transfection of miR-208b mimics to achieve the effect of over-expression of miR-208b. **(F)** Relative expression level of MyHC after transfection of 100 nM miR-208b inhibitor and control on days 0, 2, 4 during differentiation. **(G–J)** respectively indicate relative expression levels of Myh1 **(G)**, Myh2 **(H)**, Myh4 **(I)**, and Myh7 **(J)** on 0, 2, and 4 days of C2C12 myogenic differentiation after miR-208b expression is inhibited. Statistical comparisons are performed using multiple t-tests.*p < 0.05.

C2C12 cells were used as a model of myogenic differentiation to simulate the process of muscle fiber formation *in vitro*. Changes in expression levels of different muscle fiber marker genes MyHC and miR-208b during myogenic differentiation were monitored and detected (Figures 2A–E). To clarify the specific functions of miR-208b in the transformation of different types of muscle fibers, miR-208b mimics were transfected into C2C12 myoblasts (Figure 2F). After successfully overexpressing miR-208b, myogenic differentiation of C2C12 cells was induced and changes in the expression levels of different types of MyHC at the mRNA level were detected (Figures 3A–D). Results showed that after increasing the expression of miR-208b, the expression of the slow muscle marker gene Myh7 increased, while the expression of the fast muscle marker gene Myh4 decreased significantly. The expression of intermediate muscle fiber marker genes Myh1 and Myh2 also showed a decreasing trend. On the other hand, following the transfection with miR-208b inhibitors, the opposite results were obtained (Figures 3F–J), further indicating that miR-208b can regulate the conversion of fast-twitch fibers to slow-twitch fibers. At the same time, we also observed changes in protein expression of Myh4, Myh7, and MYL9 which is enriched in fast-twitch fibers. The results showed that the expression of Myh4 protein decreased significantly, while the expression of Myh7 protein increased significantly, which is consistent with the transcription level results (Figure 3E). The expression level of MYL9 was significantly reduced (Figure 3E), indicating that the overexpression of miR-208b may affect the expression of genes related to fast-twitch fiber formation.

### MiR-208b Inhibits Expression of Target Gene Mettl8 by Binding to 3′UTR

**FIGURE 4:**
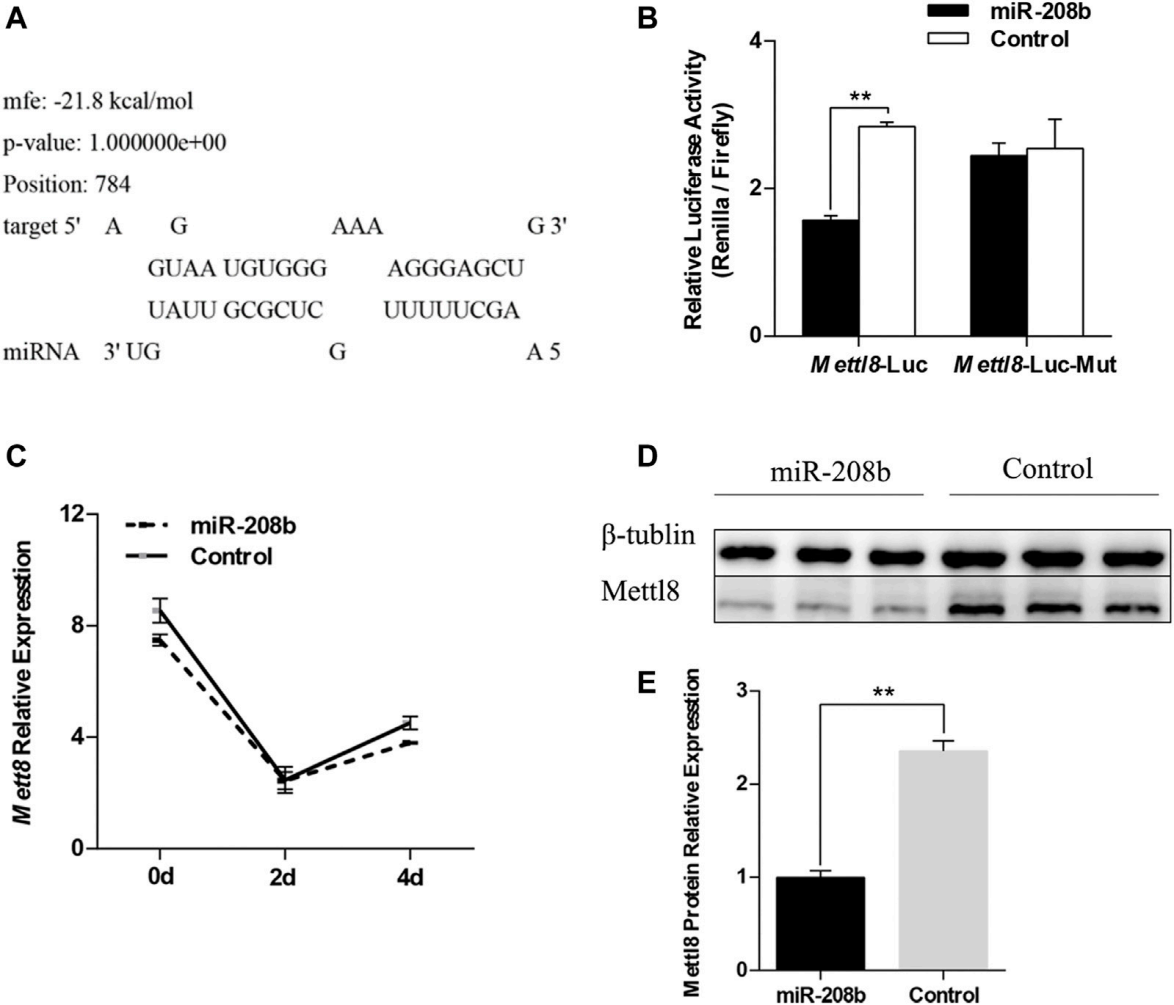
Verification of the targeting relationship between miR-208b and Mettl8. **(A)** The binding site of miR-208b and Mettl8 3′UTR sequence. **(B)** Results of dual luciferase reporter assay. Mettl8-Luc: wild-type plasmid; Mett18-Luc-Mut: Mettl8 mutant plasmid. The ordinate is the relative luciferase activity (renilla luciferase activity/firefly luciferase activity). MiR-208b represents C2C12 cells transfected with miR-208b mimics to achieve the effect of miR-208b over-expression. **(C)** Changes in Mettl8 mRNA expression level after miR-208b overexpression in C2C12 cells during myogenic differentiation. **(D)** Western blot results of Mettl8 protein after miR-208b overexpression. **(E)** Relative expression level of Mettl8 protein calculated using Image J software. Statistical comparisons are performed using multiple *t*-tests in **(B–C)** and using unpaired *t*-tests in **(E)**. **p < 0.01.

We used RNAhybird (Rehmsmeier et al., 2004) to predict the binding site of miR-208b in the target gene Mettl8 (Figure 4A). To verify the targeting relationship between miR-208b and Mettl8, miR-208b mimics and luciferase vectors were co-transfected in HEK293T cells. The luciferase vectors contained wild type Mettl8 3′UTR sequence or sequence with a point mutation at binding site. We observed that compared with negative control, the luciferase activity of the cells co-transfected with miR-208b and Mettl8 3′UTR sequence was significantly decreased (Figure 4B), while the luciferase activity in the cells transfected with mutant sequence did not change significantly (Figure 4B), indicating that miR-208b can target to bind Mettl8 and inhibit its expression. We then further explored the effect of overexpressing miR-208b on mRNA level and the protein level of Mettl8 in C2C12 cells. RT-qPCR results showed that after overexpression of miR-208b, the expression of Mettl8 mRNA level in C2C12 cells was not significantly reduced [(Figure 4C), but the protein level was significantly reduced (Figures 4D,E)], indicating that miR-208b targeted to Mettl8 3′UTR sequence by incomplete complementary pairing to inhibit its protein translation process.

### Mettl8 Promotes the Conversion of Slow Muscle Fibers to Fast Muscle Fibers in the Process of C2C12 Myogenic Differentiation

**FIGURE 5:**
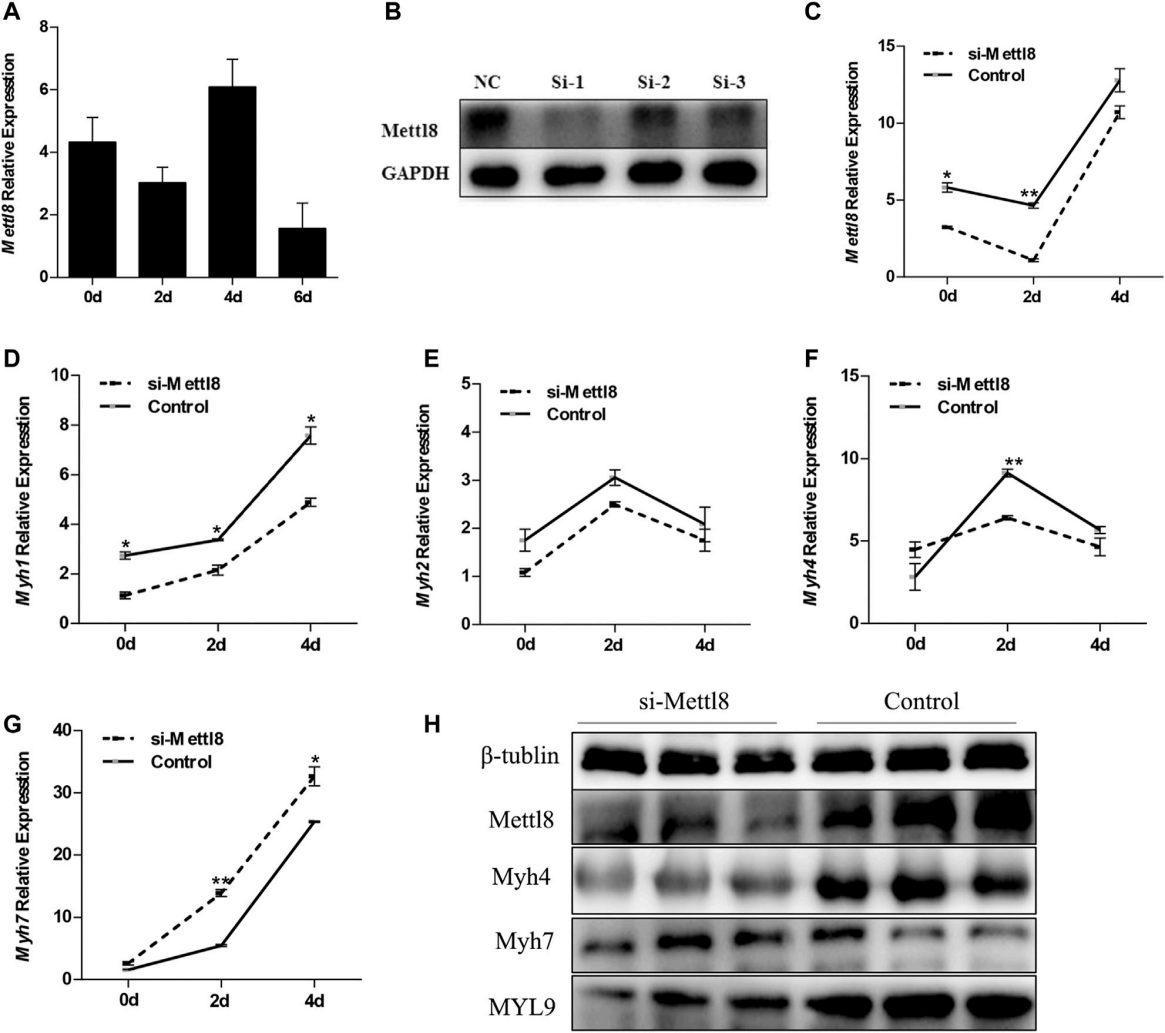
The effect of knocking down Mettl8 on MyHC expression in C2C12 cells during myogenic differentiation. **(A)** The expression of Mettl8 in the process of myogenic differentiation of C2C12 cells. **(B)** The change of Mettl8 protein expression level after transfection of siRNA, NC represents negative control, si-1, si-2, and si-3 represent the three designed Mettl8 interfering RNA. **(C)** Changes in Mettl8 mRNA expression after transfection of siRNA. **(D–G)**: Expression levels of Myh1 **(D)**, Myh2 **(E)**, Myh4 **(F)**, and Myh7 **(G)** in the endogenously Mettl8 knockdown C2C12 cells during the myogenic differentiation on days 0, 2, 4. **(H)** Protein expression levels of Mettl8, Myh4, Myh7, MYL9 in the endogenously Mettl8 knockdown C2C12 cells. si-Mettl8: C2C12 cells transfected with 50 nM Mettl8 interfering RNA to reduce the expression of endogenous Mettl8. Statistical comparisons are performed using ordinary one-way ANOVA in **(A)** and using multiple *t*-tests in **(D–G)**. *p < 0.05; **p < 0.01.

Once the targeting relationship between miR-208b and Mettl8 was determined, we then monitored the expression pattern of Mettl8 during myogenic differentiation (Figure 5A). To further explore the effect of Mettl8 on muscle fiber transformation, RNAi technology was used to knock down the expression of C2C12 endogenous Mettl8 protein (Figures 5B,C), followed by using RT-qPCR to analyze the expression of different types of muscle fiber type marker genes MyHC. The test results showed (Figures 5D–G) that after down regulated Mettl8 expression, Myh4 expression was significantly down-regulated on the second day of differentiation, Myh7 expression was significantly up-regulated on 2–4 days of differentiation. Although Myh1 and Myh2 were downregulated within 4 days of differentiation. To further confirm the translation level, Western blot was used to detect the protein expression of Myh4, Myh7 and MYL9 in the cells on the 4th day of differentiation. The results showed (Figure 5H) that the expression level of Myh4 and MYL9 was significantly reduced while expression level of Myh7 increased significantly following the inhibition of Mettl8 protein expression. These results are consistent with those obtained with transfection of miR-208b mimics and further demonstrated that Mettl8 promoted the conversion of slow muscle fibers to fast muscle fibers during the process of C2C12 myogenic differentiation.

### Effect of MiR-208b and Mettl8 on the Transformation of Different Types of Muscle Fibers in Mouse Skeletal Muscle

**FIGURE 6:**
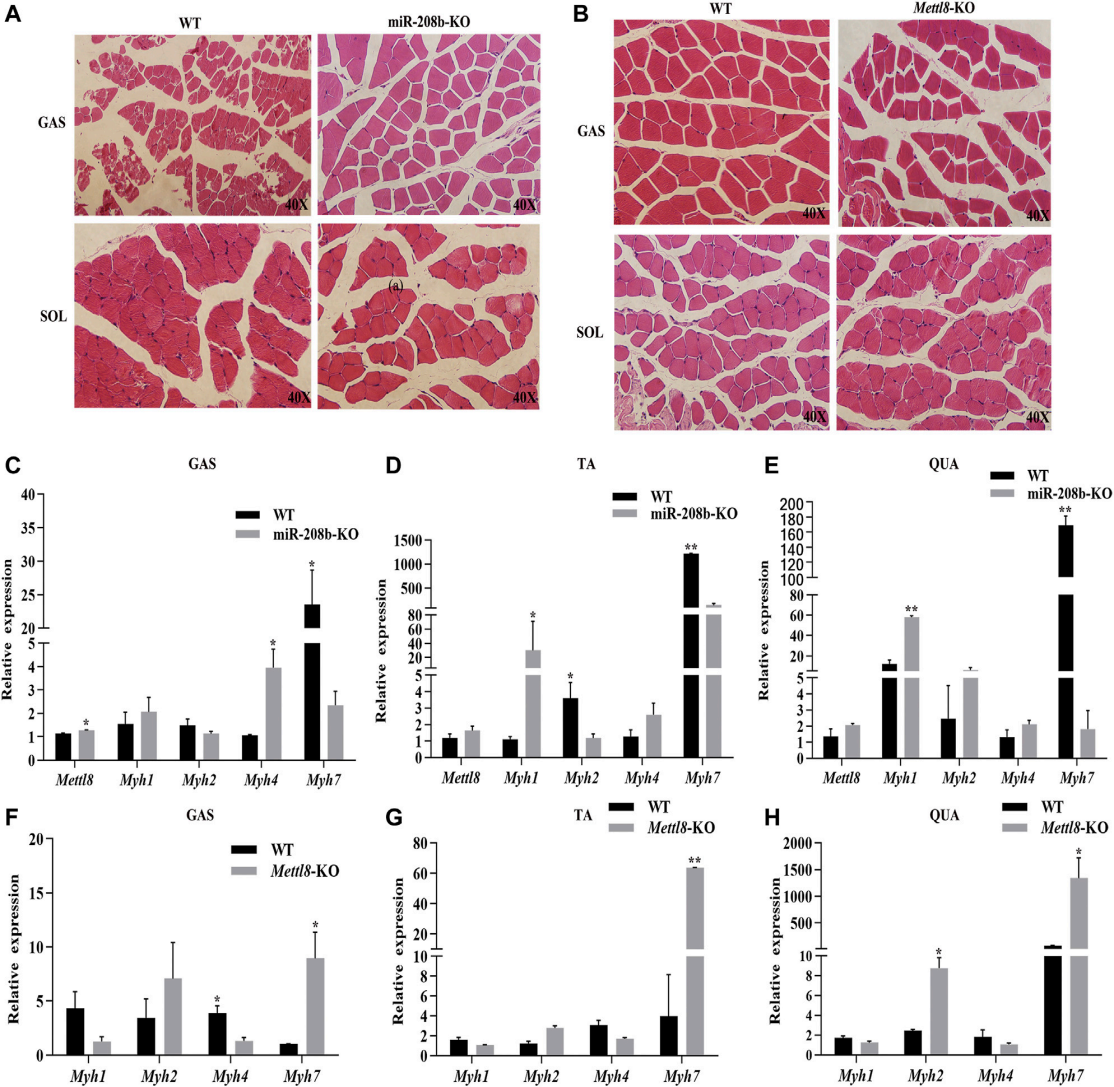
Transformation of different skeletal muscle fiber types in miR-208b and Mettl8 knockout mice. **(A)** HE staining (40×) of skeletal muscle from miR-208b knockout mice and wild-type mice. **(B)** HE staining (40×) of skeletal muscle from Mettl8 knockout mice and wild-type mice. **(C–E)**: Expression levels of Mettl8 and MyHC in GAS **(C)**, TA **(D)**, and QUA **(E)** of miR-208b knockout mice and wild-type mice, respectively. **(F–H)**: Expression levels of Mettl8 and MyHC in GAS **(F)** TA **(G)** and QUA **(H)** of Mettl8 knockout mice and wild-type mice, respectively. WT: wild-type mice; miR-208b-KO: miR-208b gene knockout mice; Mettl8-KO: Mettl8 gene knockout mice; GAS: gastrocnemius muscle; SOL: soleus muscle; QUA: quadriceps muscle. Statistical comparisons are performed using multiple *t*-tests. *p < 0.05; **p < 0.01.

To further explore the functions of miR-208b and Mettl8 *in vivo*, we produced miR-208b and Mettl8 gene knockout mice using CRISPR/Cas9 technology to examine if miR-208b and Mettl8 gene knockout has any effect on muscle fiber morphology. We isolated gastrocnemius and soleus muscles from 8-week-old knockout mice and wild-type mice and performed HE staining. It was observed that, compared with wild type mice, gastrocnemius muscle fibers became larger in miR-208b knockout mice (Figure 6A). On the other hand, there was no significant changes in soleus muscle, which contained more slow muscle fibers (Figure 6A). Compared with wild-type mice, muscle fibers in gastrocnemius muscle of Mettl8 gene knockout mice became smaller, indicating that the muscle fibers may be transformed into thinner and longer slow muscle fibers (Figure 6B). Like observed in miR-208b knockout mice, there is no significant changes in muscle fibers of soleus muscle in Mettl8 knockout mice (Figure 6B).

We also used RT-qPCR to determine the expression levels of different muscle fiber marker genes MyHC in mouse skeletal muscle. The results showed (Figures 6C–E) that, compared with wild-type mice, the expression level of Myh7 in GAS, TA, and QUA decreased significantly. On the other hand, the expression level of the target gene Mettl8 increased in all three skeletal muscles, being significantly up-regulated in GAS. The expression of Myh1 increased in all three skeletal muscles, reaching to significant level in TA and QUA. The expression of Myh2 decreased in GAS and TA muscle but increased in QUA muscle, reaching a significant decrease level in TA. Myh4 expression increased in all three skeletal muscles, but its change is significant in GAS. Compared with wild-type mice, the expression levels of Myh7 in GAS, TA and QUA increased significantly in Mettl8 gene knockout mice. The expression level of Myh1 in these three skeletal muscles from Mettl8 gene knockout mice decreased, but the decrease was not significant. The expression level of Myh2 increased in these three skeletal muscles from Mettl8 gene knockout mice, and the increase reached a significant level in QUA. The expression level of Myh4 was down-regulated in these three skeletal muscles from Mettl8 gene knockout mice, with significant changes in GAS (Figures 6F–H). The above experimental results prove that miR-208b and Mettl8 play the opposite effect in the conversion of fast and slow muscle fibers following their gene knocking out, but the degree of their influence is slightly different in different types of skeletal muscles, likely due to the different composition ratios of different types of skeletal muscles.

## Discussion

MiRNA is a short single-stranded RNA that does not encode protein. It has the function of regulating targeted gene expression at the translation level or transcription level. (Filipowicz, 2005; Pu et al., 2019). With the advancement of high-throughput sequencing technology, more and more miRNAs have been identified to be involved in regulating a variety of biological processes, including skeletal muscle growth and development (O’rourke et al., 2007). MA et al. extracted RNAs from skeletal muscles such as peroneus longus, longissimus dorsi, and psoas major in pigs, and identified different types of differential miRNAs from different types of muscles through transcriptome sequencing (Ma et al., 2015). They speculated that miRNAs may be involved in the regulation of muscle fiber type transformation. Liu et al. observed that an individual knockout of miR-133a-1 or miR-133a-2 did not significantly affect the growth and development of mouse muscles, but simultaneous knockout of both miR-133a-1 and miR-133a-2 led to disease of fast-twitch fibers, mitochondrial damage, and the conversion of fast and slow-twitch fibers (Liu et al., 2008). Wang et al. noticed that the expression of miR-499 in the pig’s extensor toe and soleus muscle was negatively correlated with the expression of the key transcription factor Sox6 (SRY-box transcription factor 6) of fast-contracting muscle fibers (Wang et al., 2017). After overexpression of miR-499 in porcine muscle satellite cells, the expression of MyHC I and MyHC IIA mRNA increased. MSTN is a negative regulator of skeletal muscle growth and development. It is involved in complex cellular signaling pathways and inhibits myogenesis by regulating the expression of target genes and other molecular mechanisms (Cai et al., 2017), but the underlying molecular mechanism of MSTN functions has is still not fully understood yet. Our previous studies showed that the expression level of miR-208b decreased significantly in MKO Meishan pigs, indicating that miR-208b may be involved in regulating skeletal muscle growth and development. Our current study further confirmed the reliability of the previous miRNA sequencing results through molecular and biological experiments. Our results demonstrated that the expression level of miR-208b in the skeletal muscle of MKO Meishan pigs was significantly down-regulated, indicating that miR-208b is involved in the regulation of skeletal muscle growth and development. The proportion of slow-twitch fibers was higher on 65 days of embryonic development than post birth in pigs. Our results showed that miR-208b is highly expressed on 65 days of embryonic development, which suggested that miR-208b was involved in the formation of slow-twitch muscle fibers. We speculate that MSTN may act as an upstream regulator that affects the processing of miR-208b precursor sequence or the transcription of its coding sequence, which is different from the results of MSTN as a downstream target gene of miRNA reported in a previous report (Callis et al., 2009). Our results may provide a new insight to study the mechanism of MSTN action in skeletal muscle growth and development.

C2C12 myoblasts are often used as an *in vitro* model to study of skeletal muscle growth and development. Many previous studies have demonstrated that some regulatory factors, including miRNA, can affect the expression of different muscle fiber type marker genes during C2C12 cell myogenic differentiation. Xu et al. showed that, during the process of C2C12 myogenic differentiation, overexpression of miR-139-5p can down-regulate MyHC I and MyHC IIA by inhibiting the expression of CaN, NFATc1, MEF2C, and MCIP1.4 in the CaN/NFAT signaling pathway. On the other hand, inhibition of miR-139-5p expression led to the opposite result (Xu et al., 2018). Cheng et al. observed that miR-204-5p can significantly reduce the ratio of slow muscle fiber genes in myoblasts by targeting MEF2C and ERR*γ* with overexpressing or inhibiting the expression of miR-204-5p in C2C12 cells post induction of myogenic differentiation (Cheng et al., 2018). In our study, liposome transfection was used to transfect miR-208b mimics and inhibitors in C2C12 myoblasts and our results confirmed that miR-208b has an effect on the expression of different muscle fiber marker genes at mRNA and protein levels during the process of myogenic differentiation. In summary, our study indicates miR-208b can regulate the transformation of fast and slow muscle fibers.

The analysis of preliminary screening sequence and bioinformatical method along with results from dual luciferase experiment confirmed that Mettl8 is a target gene of miR-208. Mettl8 is a member of the methyltransferase-like protein family and is also a tension-inducing or inhibiting protein (TIP). Mettl8, as a TIP protein, has three subtypes, Tip1, Tip2, Tip3. And these three subtypes contain nucleic acid receptor co-regulators, histone acetyltransferase, and sequence characteristics of the chromatin remodeling factor of histone deacetylase (Heery et al., 1997; Boyer et al., 2002). Previous studies demonstrated that Tip1 and Tip3 are extremely sensitive to the tension of smooth muscle. Under tension, Tip1 promotes myogenic differentiation fate, and under the influence of tension inhibition, Tip3 promotes adipogenic differentiation (Jakkaraju et al., 2005). To date, there has been no report that Mettl8 can participate in the regulation of skeletal muscle growth and development related processes. To verify the biological function of Mettl8 in muscle fiber typing, we used C2C12 cells as a model to explore the changes in Mettl8 expression during myogenic differentiation and found that its expression pattern is similar to Myh4 expression. Then we further conducted an interference experiment with Mettl8 and confirmed that Mettl8 can indeed affect the expression of fast and slow muscle marker genes at both the transcription and protein levels.

We successfully constructed miR-208b gene knockout mice and compared with wild-type mice. In terms of phenotype, there is no significant change in the morphology of muscle fibers of soleus muscle except the fact that the cross-section of single muscle cell of gastrocnemius muscle became larger. However, at the molecular level, there is a significant change in the expression levels of different muscle fiber marker genes MyHC in the skeletal muscle of knockout mice has changed significantly. With the advancement of miRNA research in muscles, it is clear that some miRNAs are found to be specifically expressed in muscles. The muscle specific miRNAs are called MyomiR, such as miR-206, miR-208b and miR-499 that are enriched in type I muscle fibers. Most knocked out MyomiRs in mice have little effect on the phenotype of skeletal muscle (Zhao et al., 2007; Boettger et al., 2014). For example, skeletal muscle-specific knockout of miR-206 did not result in an obvious change in phenotype as evidenced by no significant changes in body weight, soleus muscle weight, or the morphology of muscle fibers (Williams et al., 2009). Additionally, we also generated Mettl8 knockout mice and compared with wild-type mice. Again, there is no obvious change in phenotype except for the increase of gastrocnemius fiber area. At the molecular level, the pattern of change in MyHC expression is just the opposite of miR-208b knockout. This clearly shows that Mettl8 is the target gene of miR-208b and has the opposite effect on muscle fiber transformation compared to miR-208b. We observed that miR-208b regulates the conversion of muscle fibers to slow muscle by targeting and inhibiting Mettl8 while Mettl8 can in turn affect the host gene Myh7 encoding miR-208b, thus forming a regulatory network which is similar to the regulatory network of miR-499 and its target gene Sox6 in a previous study using a mouse model of skeletal muscle atrophy (Mccarthy et al., 2009). Of course, there may be many regulatory factors and signal pathways similar to MSTN involved in this network, and the specific mechanism needs to be investigated in depth in the future.

In this study, we used MTSN-edited Meishan pigs as research animals to successfully confirm the previous sequencing analysis results of miR-208b and Mettl8 and then analyzed expression profiles of miR-208b and Mettl8 in skeletal muscle from different tissues during embryonic development and post birth. Then we employed C2C12 myoblasts as a model to investigate the effect of overexpression or inhibition of miR-208b on the expression of different marker genes MyHC during the differentiation of myoblasts. Results with C2C12 myoblasts show that miR-208b can promote the production of slow muscle fibers. Use of bioinformatic analysis and dual luciferase experiments further verified the targeting relationship between miR-208b and Mettl8. Through the Mettl8 interference experiment in C2C12 cells, it was confirmed that Mettl8 can affect the differentiation of different marker genes MyHC during myoblast differentiation and its effect is just the opposite of miR-208b. Finally, the miR-208b and Mettl8 gene knockout mice were successfully generated and results from these knockout mice further demonstrated that miR-208b and Mettl8 play opposite roles in the transformation of fast and slow muscle fibers.

## Data Availability

The original contributions presented in the study are included in the article/Supplementary Material, further inquiries can be directed to the corresponding author.
