# Supplementary material for: MiR-208b Regulates the Conversion of Skeletal Muscle Fiber Types by Inhibiting Mettl8 Expression
Source: Front Genet. 2022 Feb 23;13:820464. doi: 10.3389/fgene.2022.820464 (PMC8905228; doi:10.3389/fgene.2022.820464)
Supplement: Supplementary file 14 [file DataSheet1.docx]

Table S1. Reverse transcription and RT-qPCR primer sequences

| Primer name | Primer sequence（5’ → 3’） |
| --- | --- |
| SSC-miR-208b RT | TCGTATCCAGTGCAGGGTCCGAGGTATTCGCACTGGATACGACACAAAC |
| Ssc-miR-208b F | GCGCGATAAGACGAACAAAAG |
| Ssc-miR-208b R | AGTGCAGGGTCCGAGGTATT |
| Ssc-U6 RT | TTCACGAATTTGCGTGTCAT |
| Ssc-U6 F | CGCTTCGGCAGCACATATAC |
| Ssc-U6 R | TTCACGAATTTGGGTGTCAT |
| Ssc-Mettl8 F | TTGCTTCTGGAGCTGTGGAACTAG |
| Ssc-Mettl8 R | TCACAACACCTTGCATCCTGTCAG |
| TBP F | AACAGTTCAGTAGTTATGAGCCAGA |
| TBP R | AGATGTTCTCAAACGCTTCG |
| Mmu-miR-208b RT | GTCGTATCCAGTGCAGGGTCCGAGGTATTCGCACTGGATACGACACATAA |
| Mmu-miR-208b F | CGAAGCTTTTTGCTCGCG |
| Mmu-miR-208b R | AGTGCAGGGTCCGAGGTATT |
| U6 RT | CGCTTCACGAATTTGCGTGTCAT |
| U6 F | GCTTCGGCAGCACATATACTAAAAT |
| U6 R | CGCTTCACGAATTTGCGTGTCAT |
| Mettl8 F | CCTGGATGTCGTTCTCCTTGTC |
| Mettl8 R | GGCTTCAGCAACCTGGACAG |
| Myh1 F | GCGACAGACACCTCCTTCAAGAAC |
| Myh1 R | CCAGCCAGCCAGCGATGTTG |
| Myh2 F | GCGACAGACACCTCCTTCAAGAAC |
| Myh2 R | GTCCAGCCAGCCAGTGATGTTG |
| Myh4 F | CAATCAGGAACCTTCGGAACAC |
| Myh4 R | GTCCTGGCCTCTGAGAGCAT |
| Primer name | Primer sequence（5’ → 3’） |
| Myh7 R | ATGTTCTCTTTCAGGTCGTCAT |
| GAPDH F | GTGCCGCCTGGAGAAACCT |
| GAPDH R | AAGTCGCAGGAGACAACC |

Ssc: Sus scrofa

Mmu: Mus musculus

Table S2. PCR primer sequences

| Primer name | Primer sequence（5’ → 3’） |
| --- | --- |
| Mettl8 F | GGCGCTCGAGCATTCACTGCATTCTCAGGAC |
| Mettl8 R | AATGCGGCCGCTTTTGGAAGAAAAGGTTTATTTTAG |
| Mettl8-mut F | TGGGAAAACCCTCGAGAGCACTGGCACACCAGT |
| Mettl8-mut R | FCAGTGCTCTCGAGGGTTTTCCCACACTTACTTG |

Mettl8-mut: mutated binding site on Mettl8 for miR-208b
